# 2-Bromo-2-(5-bromo-1*H*-1,2,4-triazol-1-yl)-1-(2,4-difluoro­phen­yl)ethanone

**DOI:** 10.1107/S1600536810011359

**Published:** 2010-03-31

**Authors:** Kun Wan, Bo Fang, Guang-Zhou Wang, Cheng-He Zhou

**Affiliations:** a School of Chemistry and Chemical Engineering, Southwest University, Chongqing 400715, People’s Republic of China

## Abstract

In the title compound, C_10_H_5_Br_2_F_2_N_3_O, the mean planes of the benzene and triazole rings form a dihedral angle of 84.86 (2)°. In the crystal structure, weak inter­molecular C—H⋯O hydrogen bonds link mol­ecules into extended chains propagating along the *c* axis.

## Related literature

For general properties of 1,2,4-triazole derivatives, see: Garfunkle *et al.* (2008 ▶); Yu *et al.* (2009 ▶). For their anti­microbial activity, see: Luo *et al.* (2009 ▶); Zhang *et al.* (2010 ▶).
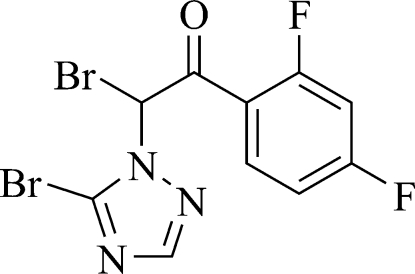


## Experimental

### 

#### Crystal data

C_10_H_5_Br_2_F_2_N_3_O
*M* *_r_* = 380.99
Monoclinic, 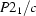

*a* = 9.273 (2) Å
*b* = 9.375 (2) Å
*c* = 14.982 (3) Å
β = 104.916 (3)°
*V* = 1258.5 (5) Å^3^
*Z* = 4
Mo *K*α radiation
μ = 6.46 mm^−1^
*T* = 298 K
0.26 × 0.25 × 0.25 mm

#### Data collection

Bruker SMART CCD diffractometer
Absorption correction: multi-scan (*SADABS*; Sheldrick, 1996 ▶) *T*_min_ = 0.199, *T*_max_ = 0.203
6096 measured reflections
2206 independent reflections
1577 reflections with *I* > 2σ(*I*)
*R*_int_ = 0.036

#### Refinement

*R*[*F* ^2^ > 2σ(*F* ^2^)] = 0.039
*wR*(*F* ^2^) = 0.100
*S* = 1.04
2206 reflections
163 parameters
H-atom parameters constrained
Δρ_max_ = 0.61 e Å^−3^
Δρ_min_ = −0.49 e Å^−3^

### 

Data collection: *SMART* (Bruker, 2001 ▶); cell refinement: *SAINT-Plus* (Bruker, 2001 ▶); data reduction: *SAINT-Plus*; program(s) used to solve structure: *SHELXS97* (Sheldrick, 2008 ▶); program(s) used to refine structure: *SHELXL97* (Sheldrick, 2008 ▶); molecular graphics: *SHELXTL* (Sheldrick, 2008 ▶); software used to prepare material for publication: *SHELXTL*.

## Supplementary Material

Crystal structure: contains datablocks I, global. DOI: 10.1107/S1600536810011359/lh5016sup1.cif

Structure factors: contains datablocks I. DOI: 10.1107/S1600536810011359/lh5016Isup2.hkl

Additional supplementary materials:  crystallographic information; 3D view; checkCIF report

## Figures and Tables

**Table 1:** Hydrogen-bond geometry (Å, °)

*D*—H⋯*A*	*D*—H	H⋯*A*	*D*⋯*A*	*D*—H⋯*A*
C7—H7⋯O1^i^	0.93	2.55	3.229 (5)	130

Symmetry code: (i) 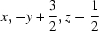.

**Fig. 1.**
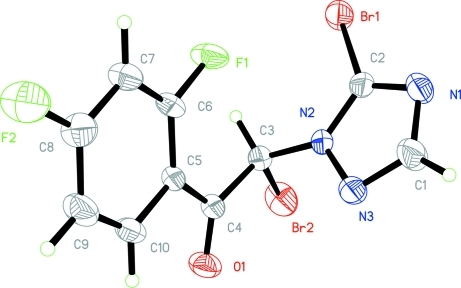
The molecular structure of (I), showing the atom-numbering scheme. Displacement ellipsoids are drawn at the 50% probability level.

## supplementary crystallographic information

### Comment

1,2,4-Triazole derivatives are important types of nitrogen-containing aromatic heterocyclic compounds with excellent safety profiles, favorable pharmacokinetic characteristics and a wide range of biological activities (Garfunkle *et al.*, 2008; Yu *et al.*, 2009). Our attention has been focused on the discovery of novel 1,2,4-triazole compounds as antimicrobial agents, and we found that some reported 1,2,4-triazole compounds display significant antimicrobial activities (Luo *et al.*, 2009; Zhang *et al.*, 2010). As part of our research research, we report herein structure of the title compound (I).

The molecular structure of the title compound is shown in Fig. 1. The mean planes of the benzene and triazole rings form a dihedral angle of 84.86 (2) °. In the crystal structure weak intermolecular C—H···O hydrogen bonds link molecules into extended chains along the c axis.

### Experimental

To a solution of 1-(2,4-difluorophenyl)-2-(1*H*-1,2,4-triazol-1-yl)ethanone (1.0 g, 4.4 mmol), sodium acetate (1.4 g, 4.4 mmol) and acetic acid (4 ml) was added the mixture of Br_2 _(0.45 ml) and acetic acid (2.5 ml) dropwise, and stirred at 338-348 K. The progress of the reaction was monitored by TLC. Upon completion, the reaction was extracted with chloroform (15 ml × 3). The filtrate was concentrated and then directly purified by chromatographic column (chloroform) to afford the title compound (I). A crystal suitable for X-ray analysis was grown from a solution of (I) in a mixture of petroleum and chloroform by slow evaporation at room temperature.

### Refinement

Hydrogen atoms were placed in calculated positions with C—H = 0.93Å and 0.98Å with U_iso_(H) = 1.2U_eq_(C)

### Crystal data

**Table:**

C_10_H_5_Br_2_F_2_N_3_O	*F*(000) = 728
*M**_r_* = 380.99	*D*_x_ = 2.011 Mg m^−^^3^
Monoclinic, *P*2_1_/*c*	Mo *K*α radiation, λ = 0.71073 Å
Hall symbol: -P 2ybc	Cell parameters from 1838 reflections
*a* = 9.273 (2) Å	θ = 2.3–22.5°
*b* = 9.375 (2) Å	µ = 6.46 mm^−^^1^
*c* = 14.982 (3) Å	*T* = 298 K
β = 104.916 (3)°	Block, colourless
*V* = 1258.5 (5) Å^3^	0.26 × 0.25 × 0.25 mm
*Z* = 4	

### Data collection

**Table:**

Bruker SMART CCD diffractometer	2206 independent reflections
Radiation source: fine-focus sealed tube	1577 reflections with *I* > 2σ(*I*)
graphite	*R*_int_ = 0.036
φ and ω scans	θ_max_ = 25.0°, θ_min_ = 2.3°
Absorption correction: multi-scan (*SADABS*; Sheldrick, 1996)	*h* = −10→11
*T*_min_ = 0.199, *T*_max_ = 0.203	*k* = −10→11
6096 measured reflections	*l* = −15→17

### Refinement

**Table:**

Refinement on *F*^2^	Primary atom site location: structure-invariant direct methods
Least-squares matrix: full	Secondary atom site location: difference Fourier map
*R*[*F*^2^ > 2σ(*F*^2^)] = 0.039	Hydrogen site location: inferred from neighbouring sites
*wR*(*F*^2^) = 0.100	H-atom parameters constrained
*S* = 1.04	*w* = 1/[σ^2^(*F*_o_^2^) + (0.0482*P*)^2^ + 0.5764*P*] where *P* = (*F*_o_^2^ + 2*F*_c_^2^)/3
2206 reflections	(Δ/σ)_max_ < 0.001
163 parameters	Δρ_max_ = 0.61 e Å^−^^3^
0 restraints	Δρ_min_ = −0.49 e Å^−^^3^

### Special details

**Table:**

Geometry. All esds (except the esd in the dihedral angle between two l.s. planes) are estimated using the full covariance matrix. The cell esds are taken into account individually in the estimation of esds in distances, angles and torsion angles; correlations between esds in cell parameters are only used when they are defined by crystal symmetry. An approximate (isotropic) treatment of cell esds is used for estimating esds involving l.s. planes.
Refinement. Refinement of *F*^2^ against ALL reflections. The weighted *R*-factor wR and goodness of fit *S* are based on *F*^2^, conventional *R*-factors *R* are based on *F*, with *F* set to zero for negative *F*^2^. The threshold expression of *F*^2^ > σ(*F*^2^) is used only for calculating *R*-factors(gt) etc. and is not relevant to the choice of reflections for refinement. *R*-factors based on *F*^2^ are statistically about twice as large as those based on *F*, and *R*- factors based on ALL data will be even larger.

### Fractional atomic coordinates and isotropic or equivalent isotropic displacement parameters (Å^2^)

**Table:**

	*x*	*y*	*z*	*U*_iso_*/*U*_eq_	
Br1	0.88429 (6)	0.61973 (5)	0.21078 (5)	0.0741 (2)	
Br2	0.65968 (6)	0.75021 (7)	0.40152 (3)	0.0777 (2)	
C1	0.7324 (6)	1.0030 (5)	0.1617 (4)	0.0783 (17)	
H1	0.7343	1.0949	0.1387	0.094*	
C2	0.7921 (5)	0.7951 (5)	0.1945 (3)	0.0512 (11)	
C3	0.5841 (4)	0.7348 (5)	0.2679 (3)	0.0426 (10)	
H3	0.5927	0.6355	0.2496	0.051*	
C4	0.4187 (5)	0.7796 (4)	0.2384 (3)	0.0426 (10)	
C5	0.3310 (4)	0.7498 (4)	0.1425 (3)	0.0375 (9)	
C6	0.3760 (5)	0.6720 (4)	0.0765 (3)	0.0424 (10)	
C7	0.2865 (5)	0.6440 (5)	−0.0092 (3)	0.0502 (11)	
H7	0.3205	0.5910	−0.0521	0.060*	
C8	0.1455 (6)	0.6970 (5)	−0.0291 (3)	0.0576 (12)	
C9	0.0924 (5)	0.7769 (6)	0.0313 (4)	0.0656 (14)	
H9	−0.0044	0.8127	0.0152	0.079*	
C10	0.1867 (5)	0.8030 (5)	0.1170 (3)	0.0553 (12)	
H10	0.1525	0.8579	0.1591	0.066*	
F1	0.5154 (3)	0.6180 (3)	0.09542 (17)	0.0645 (8)	
F2	0.0553 (3)	0.6703 (4)	−0.11300 (19)	0.0911 (10)	
N1	0.8368 (5)	0.9052 (4)	0.1564 (3)	0.0719 (13)	
N2	0.6690 (4)	0.8238 (4)	0.2223 (2)	0.0450 (9)	
N3	0.6279 (4)	0.9620 (4)	0.2011 (3)	0.0602 (10)	
O1	0.3620 (4)	0.8357 (4)	0.2925 (2)	0.0640 (9)	

### Atomic displacement parameters (Å^2^)

**Table:**

	*U*^11^	*U*^22^	*U*^33^	*U*^12^	*U*^13^	*U*^23^
Br1	0.0500 (3)	0.0474 (3)	0.1299 (5)	0.0074 (2)	0.0324 (3)	−0.0047 (3)
Br2	0.0597 (4)	0.1177 (5)	0.0494 (3)	−0.0031 (3)	0.0025 (2)	−0.0060 (3)
C1	0.075 (4)	0.046 (3)	0.133 (5)	0.008 (3)	0.063 (4)	0.018 (3)
C2	0.039 (3)	0.041 (3)	0.075 (3)	−0.001 (2)	0.016 (2)	−0.010 (2)
C3	0.039 (2)	0.044 (2)	0.045 (2)	−0.0020 (19)	0.0114 (19)	−0.0029 (19)
C4	0.039 (2)	0.042 (2)	0.051 (2)	−0.0024 (19)	0.019 (2)	−0.0017 (19)
C5	0.034 (2)	0.038 (2)	0.044 (2)	−0.0016 (18)	0.0159 (17)	−0.0005 (18)
C6	0.034 (2)	0.047 (2)	0.049 (2)	0.012 (2)	0.016 (2)	0.004 (2)
C7	0.045 (3)	0.063 (3)	0.042 (2)	0.013 (2)	0.011 (2)	−0.003 (2)
C8	0.055 (3)	0.064 (3)	0.047 (3)	0.008 (3)	0.001 (2)	−0.003 (2)
C9	0.036 (3)	0.088 (4)	0.068 (3)	0.014 (3)	0.005 (2)	−0.013 (3)
C10	0.037 (3)	0.067 (3)	0.063 (3)	0.006 (2)	0.016 (2)	−0.020 (2)
F1	0.0477 (16)	0.085 (2)	0.0583 (16)	0.0246 (14)	0.0088 (12)	−0.0174 (14)
F2	0.067 (2)	0.136 (3)	0.0549 (17)	0.030 (2)	−0.0130 (15)	−0.0225 (18)
N1	0.065 (3)	0.048 (3)	0.120 (4)	0.000 (2)	0.056 (3)	0.006 (2)
N2	0.036 (2)	0.037 (2)	0.064 (2)	−0.0055 (16)	0.0180 (17)	−0.0069 (17)
N3	0.058 (3)	0.038 (2)	0.096 (3)	0.0038 (19)	0.039 (2)	0.006 (2)
O1	0.052 (2)	0.085 (2)	0.058 (2)	0.0053 (18)	0.0201 (16)	−0.0244 (18)

### Geometric parameters (Å, °)

**Table:**

Br1—C2	1.840 (4)	C5—C6	1.378 (5)
Br2—C3	1.948 (4)	C5—C10	1.387 (6)
C1—N3	1.314 (6)	C6—F1	1.348 (4)
C1—N1	1.351 (6)	C6—C7	1.362 (6)
C1—H1	0.9300	C7—C8	1.358 (6)
C2—N1	1.297 (6)	C7—H7	0.9300
C2—N2	1.339 (5)	C8—F2	1.342 (5)
C3—N2	1.435 (5)	C8—C9	1.361 (7)
C3—C4	1.541 (6)	C9—C10	1.376 (6)
C3—H3	0.9800	C9—H9	0.9300
C4—O1	1.196 (5)	C10—H10	0.9300
C4—C5	1.483 (6)	N2—N3	1.365 (5)
			
N3—C1—N1	116.8 (4)	F1—C6—C5	119.9 (4)
N3—C1—H1	121.6	C7—C6—C5	123.7 (4)
N1—C1—H1	121.6	C8—C7—C6	117.1 (4)
N1—C2—N2	111.8 (4)	C8—C7—H7	121.5
N1—C2—Br1	125.4 (3)	C6—C7—H7	121.5
N2—C2—Br1	122.8 (3)	F2—C8—C7	118.1 (4)
N2—C3—C4	109.4 (3)	F2—C8—C9	118.7 (4)
N2—C3—Br2	110.5 (3)	C7—C8—C9	123.2 (4)
C4—C3—Br2	110.1 (3)	C8—C9—C10	117.8 (4)
N2—C3—H3	108.9	C8—C9—H9	121.1
C4—C3—H3	108.9	C10—C9—H9	121.1
Br2—C3—H3	108.9	C9—C10—C5	122.0 (4)
O1—C4—C5	120.8 (4)	C9—C10—H10	119.0
O1—C4—C3	120.2 (4)	C5—C10—H10	119.0
C5—C4—C3	119.0 (3)	C2—N1—C1	101.5 (4)
C6—C5—C10	116.2 (4)	C2—N2—N3	109.1 (4)
C6—C5—C4	127.1 (4)	C2—N2—C3	130.4 (4)
C10—C5—C4	116.7 (4)	N3—N2—C3	120.5 (3)
F1—C6—C7	116.4 (3)	C1—N3—N2	100.8 (4)

### Hydrogen-bond geometry (Å, °)

**Table:**

*D*—H···*A*	*D*—H	H···*A*	*D*···*A*	*D*—H···*A*
C7—H7···O1^i^	0.93	2.55	3.229 (5)	130

Symmetry codes: (i) *x*, −*y*+3/2, *z*−1/2.
